# A performance assessment of web-based respondent driven sampling among workers with precarious employment in Sweden

**DOI:** 10.1371/journal.pone.0210183

**Published:** 2019-01-10

**Authors:** Johanna Jonsson, Mart Stein, Gun Johansson, Theo Bodin, Susanne Strömdahl

**Affiliations:** 1 Institute of Environmental Medicine, Unit of Occupational Medicine, Karolinska Institutet, Stockholm, Sweden; 2 Centre for Infectious Disease Control, National Institute for Public Health and the Environment, Bilthoven, The Netherlands; 3 Department of Public Health, Karolinska Institutet, Stockholm, Sweden; 4 Department of Medical Sciences, Uppsala University, Uppsala, Sweden; Indiana University Purdue University at Indianapolis, UNITED STATES

## Abstract

**Objectives:**

Precarious employment (PE) is a social determinant of poor health of workers. However, this population usually lack a sampling frame, making it challenging to identify the characteristics of this group. Web-based respondent driven sampling (webRDS) recruits individuals online through the social network and can provide population estimates. This study aims to assess the performance of webRDS in a population of workers with PE.

**Method:**

WebRDS was used for recruitment and data collection in the PRecarious EMployment In Stockholm (PREMIS) study. Cross-sectional questionnaire data was collected between November 2016 and May 2017. Eligible participants were living and/or working in Stockholm County, 18–65 years old, had a personal identification number and were currently employed. WebRDS performance was assessed by the total sample size, length of recruitment chains, sample composition, sample proportions and estimated RDSII population proportions with confidence intervals.

**Results:**

The webRDS process resulted in a sample of 358 recruits and a total sample of 415 participants, recruited over 1–15 waves. Of the participating seeds and recruits, 60% and 48%, respectively, successfully recruited at least one peer. The sample composition stabilized for all variables assessed. The sample proportions and RDSII estimates differed by 1–8% and the confidence intervals included the sample proportions for all variables except one.

**Conclusions:**

WebRDS successfully recruited a sufficient sample of workers with precarious employment from which population estimates could be made. Future studies should consider implementing webRDS on a national level in order to further study this population.

## Introduction

Precarious employment (PE) is a social determinant contributing to poor health of workers and families globally [1, 2]. Several definitions have been proposed for PE. The definitions are commonly multidimensional and share many common features of employment conditions, such as temporariness, employment insecurity, wages, limited social protection and rights, and powerlessness to exercise rights [2]. In previous studies, PE has been associated with poor health outcomes, such as poor mental health, physical ill-health, reduced job satisfaction, increased prevalence of occupational injuries and increased risk of mortality [2–7]. The associations between PE and poor health outcomes may be due to the stress of living under precarious conditions in terms of maintaining work and earning, or due to health risks at work [2, 8–11]. The increased risk of occupational injuries may be related to less experience and lack of introduction and safety training at the workplaces [2, 3].

There are several concerns with studying precariously employed in Sweden. For one, workers with PE cannot be identified by using data in existing registries. Due to this a clear sampling frame is lacking. Further, there is a concern that current Swedish labour statistics and surveys produce skewed data suffering from bias [12]. National surveys such as the Labour Force Study (LFS), a population based panel survey conducted through phone interviews [13], and The Work Environment Survey, a questionnaire sent to participants in LFS [14], have high non-response rates. In 2016 and 2015, the non-response rates were 43% and 53%, respectively [15, 16]. In LFS, this rate was approximately 50% among 15–24 year-olds [15]. Further, in The Work Environment Survey, participation has been comparatively low among individuals who are young, have low levels of education, low income and a foreign background, as well as among those with temporary, part-time and self-employment [16]. Therefore, there is a need for new sampling methods when conducting research on workers with precarious employment.

Respondent-driven sampling (RDS) is a chain-referral sampling method developed for populations lacking a sampling frame and that are considered hard-to-reach [17, 18]. The sampling process begins by recruiting a number of initial participants, so called seeds. Seeds are invited to participate in the study and also given a limited number of traceable invitations to the study to distribute in their networks [18, 19]. The recruitment continues until the desired sample size is reached or until the sampling procedures dies out due to reaching an endpoint in the network [17, 20]. Due to the non-probability sampling applied in RDS, the resulting sample will not be representative of the underlying population but biased towards inclusion of seeds and recruits with large social networks [17, 19, 21]. Therefore, in order to achieve population estimates, the sample is weighted to account for the non-random way the participants were collected [17]. RDS estimators are considered asymptotically unbiased when certain assumptions underlying RDS are fulfilled [17, 21–23]. However, usually not all of these assumptions are met in RDS studies [19–21, 23, 24].

In 2015, RDS had been used in over 460 studies worldwide [25]. RDS can be implemented face-to-face or via the web (webRDS) [19]. WebRDS has previously been implemented in various populations, which include men who have sex with men in Sweden and Vietnam [24, 26], university students and young adults in the US [19, 27], and the general population in the Netherlands and Thailand [28].

WebRDS holds promise for recruiting precariously employed in Sweden as internet use is high—93% of the population has internet access at home and 80% use internet every day [29]. Further, online studies provides access to the study at the chosen time and place of the participant, as well as it offers a high sense of confidentiality.

The aim of this paper is to assess the performance of web-based respondent driven sampling in a population of workers with precarious employment in Stockholm County, Sweden.

## Materials and methods

### Study design and study population

The current study is a web-based cross-sectional study conducted within the frame of the longitudinal research project PRecarious EMployment in Stockholm (PREMIS). PREMIS aims at studying health outcomes of PE among residents in Stockholm County. Inclusion criteria for participants were: holding a precarious employment at the time of participation, living and/or working in Stockholm County, being 18–65 years of age, holding and indicating a Swedish personal identification number, and giving informed consent. Precarious employment, in terms of current employment situation, was defined as primarily working but without a fixed, full-time, employment. In this study, this included one of the following types of employment: involuntary part-time employment, temporary employment, employed by the hour/called when needed, self-employed (because he/she can not get an employment), or working as a trainee. Exclusion criteria were: indicating an inaccurate personal identification number, having a fixed and full-time employment, being voluntarily self-employed, being a student or a pensioner.

### Web-survey design and data collection

We used a webRDS software [24, 26, 30] for peer recruitment and data collection via an online survey. The webRDS software contains a system for sending out e-mail or link-based invitations to the study. Each invitation contains a unique link to the survey, which have associated unique study ID’s. Through these ID’s, the webRDS system keeps track of the recruitment chains. The software also contains a system for sending out reminders for participation and for distributing incentives. Date of first log in to the survey, completion date, hashed IP number and type of device used to complete the survey, is also collected by the software.

The design of the web-page aimed at giving a good overview, a neutral impression and being easy to navigate. The participants could answer the survey in Swedish or English. The following sections were included in the survey: eligibility, informed consent and personal identification number, work, work environment, employment conditions, current life situation, health, background and social network information (S1 File). The survey applied the option to skip questions, except in terms of questions regarding eligibility criteria and informed consent. In order to limit the number of questions in the survey, information that could be obtained from registers (e.g., questions on education, foreign background etc.) was not included.

Eligibility was assessed on the first page and ineligible individuals could not proceed with the survey. Eligible respondents were prompted with detailed information about the study (S2 File) and written informed consent was attained by the respondent clicking “Yes” to “I understand the information given above and want to participate”, and thereafter proceeding to enter their personal identification number. Age was assessed when the participant entered their personal identification number which is based on the date of birth and four control numbers including a check-sum algorithm [31]. Opt-out took place by clicking “No, I do not want to participate”. Respondents indicating that they did not want to participate in the study were not contacted any further.

The webRDS recruitment process and survey was pilot tested with six convenience sampled participants that matched the inclusion and exclusion criteria.

### Seed recruitment

Recruitment was initiated by spreading information about the study through the reference group involved in the study and word of mouth; and through advertising in areas around Stockholm and online. Interested individuals were referred to the project website where information about the study and contact details to the study coordinator were published. Potential seeds who made contact were contacted via telephone. During the call, the study coordinator provided information about the study, ensured that the seeds matched the inclusion criteria, were motivated to participate, as well as able and willing to invite peers. Further, information on sex, age, and type of employment was assessed during the call in order to recruit seeds as diverse as possible. In order to recruit a group of successful seeds, the survey was sent to seeds evaluated to be a good fit for the study based on these assessments.

### WebRDS recruitment and sampling

Participants were asked to invite 1–4 peers from their own networks after completing the survey. Participants could send invitations via direct e-mail, indirect e-mail, link, or via WhatsApp or text message (the latter two if completing the survey on a smart phone) through the invitation feature of the webRDS software. If the participant invited four peers, and thereby generating four unique survey links and study ID’s, the webRDS system notified the participant that the maximum number of invitations were sent and thereafter no new invitations could be generated. The invitations included information on the study aim, eligibility criteria and contact information to the study coordinator. Reminders to respond to the survey were sent continuously during the data collection period. A first reminder was sent after three days via e-mail from the webRDS software. After 14 days, an e-mail reminder was sent to the recruiter of the peer. If the recruiter was a seed, the reminder was delivered via a phone call, primarily, or a text message, secondarily. A third and fourth reminder was sent after approximately 45 and 90 days, respectively. Further, reminders to invite peers were sent five days after completion to participants that invited one or no peers.

The study applied a dual incentive system, as is customary in RDS methodology [18]. Participants received a small economical reimbursement for participating in the survey and when two of their invited peers participated in the survey. The compensations were either a cinema ticket or a gift card worth 100 Swedish kronor (SEK; approximately 11 USD). These compensations were decided upon through thorough discussion with the reference group and thereafter piloted.

### Data cleaning and analysis

All submitted surveys were assessed in terms of eligibility criteria, completeness and suspected cheating (repeated participation). Ineligible participants and participants re-using or giving an incorrect personal identification number [31] were excluded. If two or more surveys contained the same personal identification number, the survey(s) submitted at the later point in time were disregarded. Suspected cheating was assessed by comparing the date and time of participation among peers invited by the same recruiter. Participants were further investigated if there was <30 minutes between one peer completing the survey and the other starting the survey. These participants were excluded if they shared features implying repeated participation, such as having similar e-mail addresses, using the same browser, device and operating system in several following waves of the recruitment tree. Ineligible participants are displayed in the recruitment trees, but otherwise removed from the main analyses.

WebRDS performance was evaluated by assessing the recruitment dynamics and sample characteristics. The previous was done by reporting the total number of participants, number of waves in the recruitment chains, and median network degree; plotting the cumulative sample; recruitment homophily; and assessing the stabilization of sample composition with every 25-participant increase in sample size, i.e., equilibrium. Network degree is the number of individuals that the participant is connected to in the target population [19]. This was assessed by the question “How many people whom are also precarious workers (working but without a fixed, full time employment), older than 18 years, could you invite to this study via internet if the invitations were not limited to four?”. Recruitment homophily is the ratio of the number of recruits with the same characteristic as their recruiter to the number expected by chance (i.e., 1 indicates no homophily) [32]. Equilibrium was considered reached at the point when the change in sample composition was 2% or less over five consecutive 25-participant increases. Sample characteristics were summarized in terms of sample proportions, estimated RDSII population proportions and confidence intervals [23]. Seeds were included in the full sample and analyses if not otherwise stated. Chi square analyses and Mann-Whitney U tests were conducted in order to compare recruiting and non-recruiting seeds. Recruitment homophily was assessed both for the eligible recruits and eligible recruits plus ineligible recruits in order to detect potential differences. Response rate was defined as the number of entries to the survey divided by the number of invitations sent out by the system (i.e., seed and peer invitations) during the data collection period. Non-response rate was defined as 1—response rate. Analyses were conducted using SPSS version 24 for Mac (IBM SPSS Statistics for Windows, Version 23. Armonk, NY: IBM Corp), RStudio version 1.1.383 for Mac (RStudio Inc, Inc., Boston, MA) and RDS-Analyst version 0.42 for Windows (Los Angeles, CA).

### Ethical considerations

The study was approved by the regional ethics board of Stockholm (dnr: 2016/1291-31/5). Written informed consent was attained by the respondent clicking “Yes” to the question “I understand the information given above and want to participate” after reading the study information. Data was stored on password protected encrypted servers. Personal identification numbers were replaced by serial numbers and stored separately after data collection.

## Results

**Fig 1:**
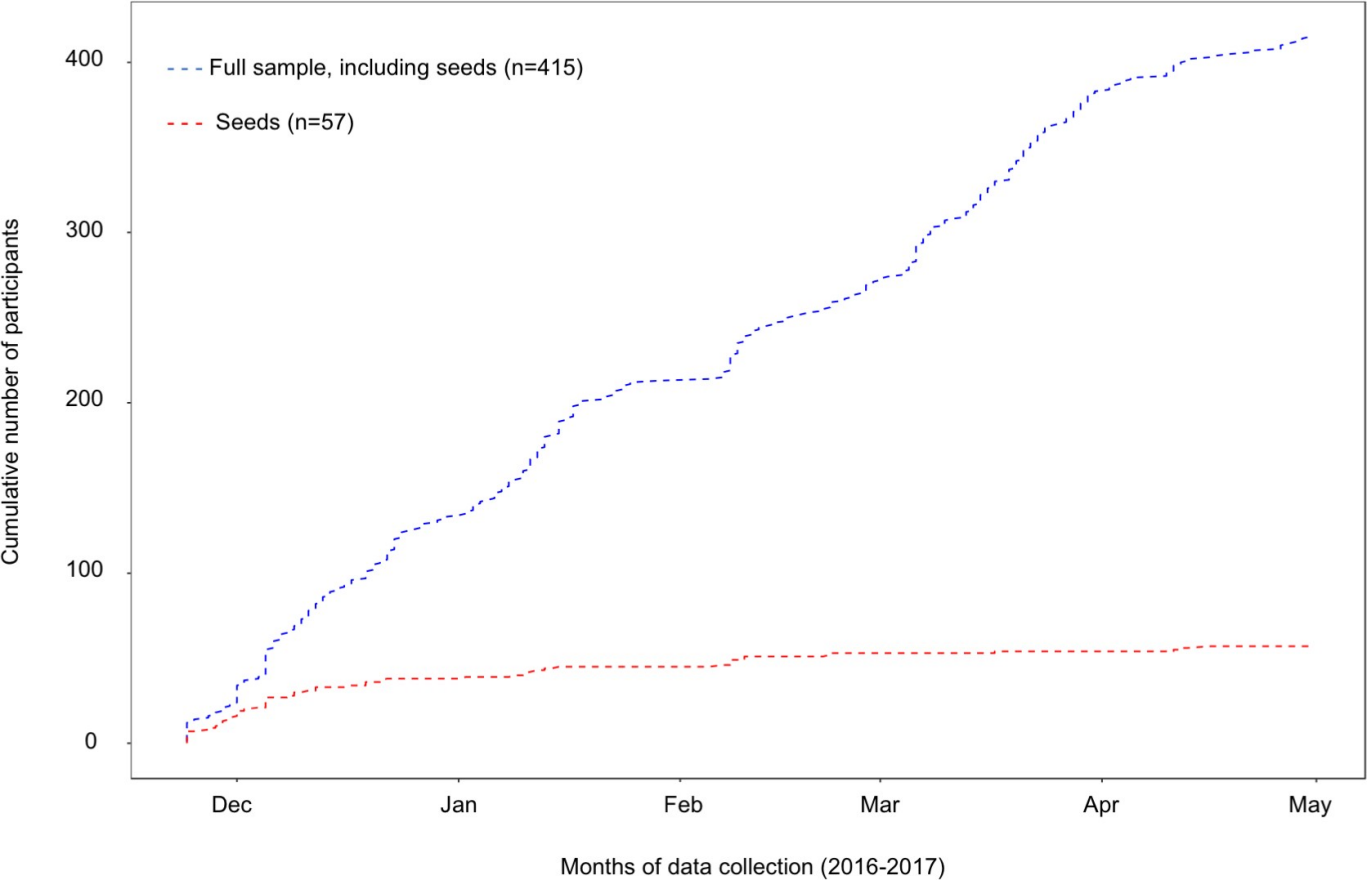
Cumulative number of participants and seeds over the course of data collection.

Data was collected between the 24^th^ of November 2016 and the 1^st^ of May 2017. A total of 75 seeds were invited continuously, out of which 57 participated. Overall, 957 invitations were sent out from the system and 595 potential participants entered the survey, giving a response rate of 62%. Participants were excluded before participation due to not matching the eligibility criteria or not completing the survey (n = 112), and after participating in the survey due to being deemed ineligible (n = 68). Of the latter, participants were excluded due to not matching employment or county (n = 6), re-using a personal number (n = 8), giving an incorrect personal number (n = 17), being underage (n = 1) and due to suspected cheating (n = 36). Participants excluded due to suspected cheating belonged to the same recruitment tree. The remaining 415 participants were considered the full sample. Fig 1 displays the cumulative number of participants over the course of data collection. After approximately one and three months of data collection, respectively, 133 and 271 participants had completed the survey.

### Seed recruitment performance

Seed characteristics and recruitment performance are displayed in Table 1. Out of the participating seeds, 34 (60%) successfully recruited at least one peer (hereafter referred to as recruiting seeds). Recruiting seeds, compared to non-recruiting seeds, were to a larger extent female (79% versus 65%), older (85% versus 61% were above 25 years) and recruited by online advertisement (71% versus 44%). Further, recruiting seeds invited two or more peers to a greater extent than non-recruiting seeds (94% vs. 63%).

**Table 1:** Descriptives and recruitment characteristics of seeds, stratified by recruiting and non-recruiting seeds.

	Recruiting seeds (N = 34)		Non-recruiting seeds (N = 23)		
	N	%	N	%	
**Sex**					
Female	27	79.4	15	65.2	
Male	7	20.6	8	34.8	0.36^b^
**Age**					
<24	5	14.7	9	39.1	
25–29	11	32.4	4	17.4	
>30	18	52.9	10	43.5	0.09^b^
**Employment type**					
Temporary	15	44.1	7	30.4	
By the hour	16	47.1	14	60.9	
Other^a^	3	8.8	2	8.7	0.57^c^
**Recruitment Method**					
Online advertisement	24	70.6	10	43.5	
Facebook	1	2.9	1	4.3	
Through unions	0	0.0	3	13.0	
Reference Group	2	5.9	3	13.0	
Incoming e-mail	6	17.6	4	17.4	
Research group	1	2.9	1	4.3	
Referral from ineligible participant	0	0.0	1	4.3	0.12^c^
**No. of reminders sent to seed**					
0	27	79.4	16	69.6	
1	4	11.8	6	26.1	
≥2	3	8.8	1	4.3	0.49^c^
**No. of peer invitations sent by seed**					
0	0	0.0	4	17.4	
1	2	5.9	4	17.4	
≥2	32	94.1	15	62.5	<0.01^c^
**No. of recruited peers by seed**					
0	0	0.0	23	100.0	
1	8	23.5			
≥2	26	76.5			<0.01^c^

^a^Self-employed, interns, and part-time employees

^b^Pearson chi square

^c^Fisher’s exact test

**Fig 2:**
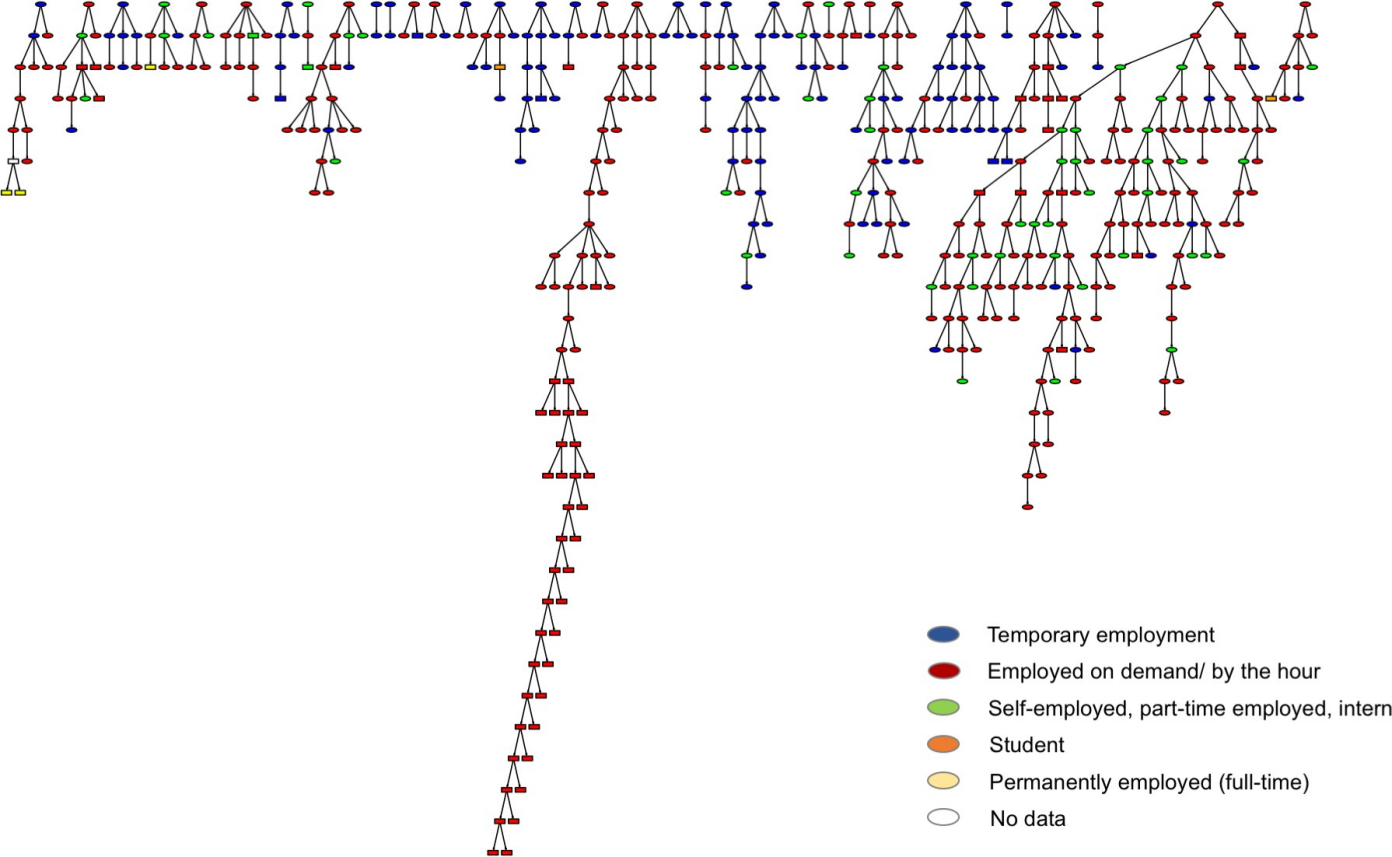
Recruitment networks showing employment type of submitted surveys. Squares indicate that the submitted survey is ineligible.

The length of the recruitment chains varied between 0–27 waves (Fig 2) with a median of four waves. Disregarding the ineligible participants, the longest recruitment chain was 15 waves and with a median of three waves. The largest recruitment tree contained 133 eligible recruits, i.e., 37% of all eligible recruits.

### Sample recruitment performance

Direct e-mail invitation and link invitation were the most popular invitation-methods (Table 2). These methods were used by 79% and 17% of the full sample, and 76% and 20% of the recruits. Out of the full sample, 58% invited two or more peers to participate in the survey and 50% successfully recruited at least one peer. Out of the recruits, 55% invited two or more peers and 48% successfully recruited at least one peer. For seeds, these proportions were 83% and 60%, respectively. The median network degree was three (range 1–300) for both the full sample and the recruits. Seeds had a median network degree of four (range 1–100). The majority of the full sample, recruits and seeds stated that 60–100% of their network degree was in the same age group (+/- 5 years). Recruits were mainly invited by a close friend or a co-worker.

**Table 2:** Recruitment and social network characteristics of full sample, stratified by recruits and seeds.

	Full sample (N = 415)		Recruits (N = 358)		Seeds (N = 57)	
	N	%	N	%	N	%
**Invitation method**						
Direct e-mail	329	79.3	272	76	57	100
Indirect e-mail	1	0.2	1	0.3	0	0.0
Link	70	16.9	70	19.6	0	0.0
Text message /Whatsapp	15	3.6	15	4.2	0	0.0
**No. of reminders sent**						
0	349	84.1	306	85.5	43	75.4
1	52	12.5	42	11.7	10	17.5
≥2	14	3.4	10	2.8	4	7.1
**Participation day**						
Mon	70	16.9	57	15.9	13	22.8
Tue	74	17.8	68	19.0	6	10.5
Wed	69	16.6	59	16.5	10	17.5
Thu	70	16.9	56	15.6	14	24.6
Fri	52	12.5	45	12.6	7	12.3
Sat	32	7.7	31	8.7	1	1.8
Sun	48	11.6	42	11.7	6	10.5
**Device type**						
Desktop	253	61.0	210	58.7	43	75.4
Smart phone	162	39.0	148	41.3	14	24.6
**No. peers invited**						
0	133	32.0	129	36.0	4	7.0
1	37	8.9	31	8.7	6	10.5
≥2	262	58.1	198	55.3	47	82.5
**No. peers participated**						
0	208	50.1	185	51.7	23	40.4
1	69	16.6	61	17.0	8	14.0
≥2	138	33.3	112	31.3	26	45.6
**% of network degree being same age**						
0%	14	3.4	10	2.8	4	7.0
1–40%	17	4.1	12	3.4	5	8.8
41–60%	42	10.1	30	8.4	12	21.1
61–100%	334	80.5	298	83.2	36	63.2
Do not know	8	1.9	8	2.2	0	0.0
**% of network degree living in the same municipality**						
0%	96	23.1	90	25.1	6	10.5
1–40%	47	11.3	41	11.5	6	10.5
41–60%	23	5.5	18	5.0	5	8.8
61–100%	226	54.5	190	53.1	36	63.2
Do not know	23	5.5	19	5.3	4	7.0
**Relation to recruiter**^**a**^						
Do not want to answer	9	2.2	7	2.0	2	3.5
Research group	34	8.2	2	0.6	32	56.1
Co-worker	44	10.6	43	12	1	1.8
Acquaintance	28	6.7	28	7.8	0	0.0
Friend	20	4.8	18	5.0	2	3.5
Close friend	177	42.7	173	48.3	4	7.0
Current partner	14	3.4	13	3.6	1	1.8
Family‎/relative	23	5.5	21	5.9	2	3.5
Close friend, co-worker	13	3.1	12	3.4	1	1.8
Close friend, acquaintance	9	2.2	9	2.5	0	0.0
Close friend, friend	6	1.4	4	1.1	1	1.8
Close friend, friend, acquaintance, co-worker	5	1.2	4	1.1	1	1.8

^a^Multiple choice possible. Only combinations with ≥ 5 respondents are shown

Among recruits, there was recruitment homophily within sex (1.34), age group (1.65), employment type (1.45), income (1.27) and hours worked per week (1.35). Results were similar when including the ineligible recruits (S1 Table).

### Sample characteristics

Slightly more than half of the sample was female (54%) and employed on demand/by the hour (59%); slightly less than half of the sample was 25–29 years old (44%) (Table 3). The majority of the sample had an income below 18 000 SEK after taxes (83%), which is the approximate median net income in Sweden [33]. Sample proportions and RDSII estimates differed by 1.3–8.1% for the full sample, and by 1–7.6% for the recruits. The sample proportions were within the estimated confidence intervals for all variables but income.

**Table 3:** Sample and population proportions (RDSII estimates) for the full sample and recruits, respectively, with 95% confidence intervals (CI’s).

	Full sample (N = 415)				Recruits (N = 358)			
	N	Sample	RDSII	95% CI's	N	Sample	RDSII	95% CI's
**Sex**								
Male	190	45.8	50.1	42.1–58.1	175	48.9	52.8	43.8–61.9
Female	225	54.2	49.9	41.9–57.9	183	51.1	47.2	38.1–56.2
**Age**								
≤24	122	29.4	33.6	25.0–42.2	108	30.2	32.6	23.7–41.5
25–29	185	44.6	43.3	35.5–51.0	170	47.5	46.3	37.6–55.0
≥30	108	26.0	23.1	15.2–31.1	80	22.3	21.1	12.9–29.3
**Employment type**								
Temporary	121	29.2	21.5	11.1–31.9	99	27.7	20.9	10.7–31.1
By the hour	243	58.6	64.2	54.6–73.8	213	59.5	64.1	54.3–73.9
Other^a^	51	12.3	14.4	8.7–20.0	46	12.8	15.1	8.6–21.5
**Income**								
<18 000 SEK	318	76.6	83.4	77.4–89.4	278	77.7	84.3	77.9–90.6
>18 000 SEK	88	21.2	13.1	7.4–18.7	72	20.1	12.5	6.4–18.7
No answer	9	2.2	3.5	0.6–6.4	8	2.2	3.2	0.6–5.8
**Average hours per week**								
≤35	309	74.5	81.7	74.3–89.1	271	75.7	82.3	74.9–89.6
>35	106	25.5	18.3	11.0–25.7	87	24.3	17.7	10.4–25.1

^a^Self-employed, interns, and part-time employees

**Fig 3:**
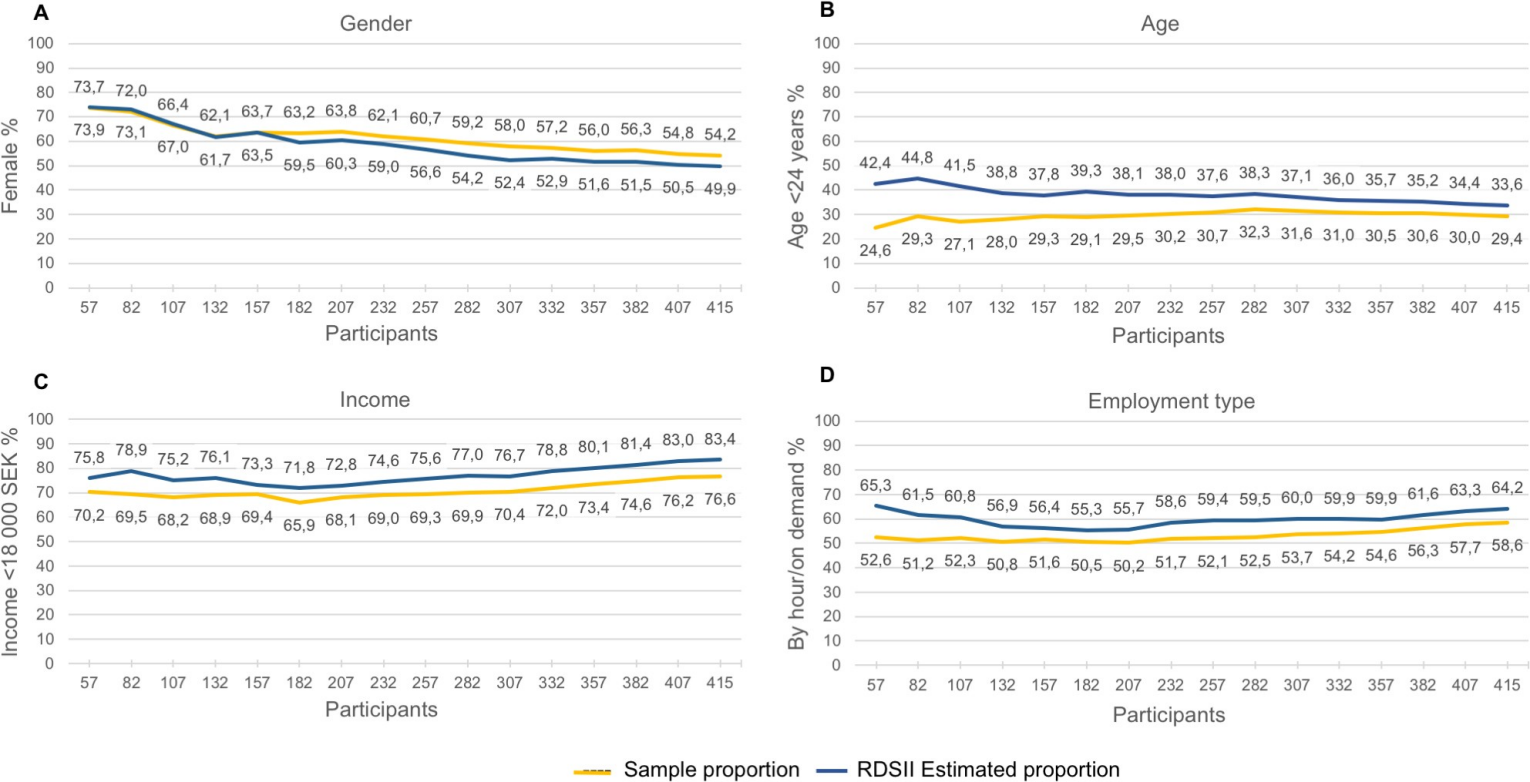
A–D. Change in sample composition with increasing sample size.

Equilibrium was reached at a cumulative number of 307 participants for sex, 157 participants for age and employment type, and 182 participants for income (Fig 3A–3D).

## Discussion

WebRDS successfully recruited a sample of workers with precarious employment. The sample was sufficiently large to make estimations, generated partly by long recruitment chains, and stabilized for all variables assessed. The sample proportions approximated the population proportions and all sample proportions except income were within the estimated confidence intervals.

WebRDS successfully captured a diverse sample of the population of precarious workers and had a response rate of 62%. However, we do not know how many of the invitations that were actually sent to an e-mail address in use, if links were generated without being sent out, or how many invitations that were read. Thereby, the response rate might actually be higher. The non-response rate was 38%, in comparison to 53% and 43% in previous national surveys assessing employment conditions and work environment [15, 16]. The comparatively lower non-response rate in this study might be due to the convenience of online recruitment and participation, compared to for example phone interviews used in LFS [12, 13]. Another benefit of RDS is the incentive system that uses a mild “peer pressure” and dual reimbursements [18, 34], which could have influenced both the willingness to invite and participate. Peer-to-peer recruitment also has the potential to reach demographic groups that are underrepresented in national surveys in Sweden, such as men, younger individuals and foreign born. The seeds managed to recruit a sample consisting of 49% men and 78% individuals under 30 years of age, indicating that these demographic groups indeed are reachable. National surveys have the benefit of being selected from a population based sampling frame and the potential of giving rise to representative samples and unbiased estimates; however, with high non-response rates among demographic groups of interest regarding precarious employment, there is a need for other data collection methods to further our understanding of the situation of this population. WebRDS could be an alternative method to successfully sample this group at a national level.

All of the variables except income had sample proportions within the estimated confidence intervals. Further, the difference between the sample and estimated proportions was greater for income compared to all the other variables. One possible explanation to these results could be that there is a large variety in income in the population of precariously employed in Stockholm. In addition, low income might skew the sample towards inclusion of low income participants as the economical reimbursement may be more attractive to participants in this subgroup [20]. A larger sample size could provide a more reliable estimate for income within the population.

Equilibrium was reached for all of the variables assessed, indicating that the sample composition became independent from the seeds [34]. The homophily results indicated in-group recruitment for all of the variables assessed. Age had the largest homophily estimate which might not be surprising as the absolute majority of the participants answered that the majority of their self-reported network degree is within the same age group. Homophily is related to equilibrium as high in-group or out-group recruitment leads to slower attainment of equilibrium [18, 35]. Thereby, it is possible that equilibrium would have been attained with even fewer participants if there were less or no homophily.

WebRDS carries several benefits in comparison with real life RDS. For instance, the ability to connect with many peers in a short time period and not having to meet in person. The population of precarious employees is young and therefore likely to use social media and have large peer-networks online. The results of this study also indicate that a large proportion of workers with a precarious employment work on the hour/on demand, which could make it challenging to recruit co-workers or friends at work face-to-face, as well as to find a suitable time to meet with an interviewer. Thereby the costs in terms of time, effort and non-anonymity are reduced. The easiness of inviting peers through multiple online options and participation being possible in short time spans via for instance smart phones is therefore likely to have facilitated the recruitment process both in terms of the number of peers the participant could invite, and the number of peers that actually participated. The online strategy was also important in terms of recruiting successful seeds, as the majority of the recruiting seeds were recruited via online advertisement.

The longest recruitment chain was 15 waves which is within the span of previous webRDS-studies reporting recruitment chains to vary between 5–24 waves [19, 24, 26, 28]. Further, 60% of the seeds and 48% of the recruits successfully recruited at least one peer, which is within the frame of results from previous studies, reporting rates between 20–90% [19, 24, 26, 28] and 52%, respectively [19]. Several factors could have influenced the recruitment success in this study. One such factor could be the attractiveness of the economical reimbursements. Of the participants, 58% invited two or more peers and 53% of these participants successfully recruited two or more peers, which indicate that the reimbursements type and value were a good choice for this population. Further, precarious employment is a situation that is related to many aspects of life, such as income and health [2, 8]. The PREMIS study might therefore be important and relevant to many of the participants, which could have increased the willingness to participate.

In this study, one recruitment chain made up 37% of the recruits. This is not uncommon in RDS studies as some seeds can be so called “super seeds”. The successfulness of super seeds can depend on a positive feedback process as the number of participants that can recruit peers increase successiviely [19]. However, certain characteristics of seeds can increase the likelihood of successful recruitment, such as ensuring sociodemographic diversity (e.g., age, sex, living area etc.), that seeds are committed to the goals of the study and have many social ties [19, 34]. In this study, motivation, as well as ability and willingness to invite peers, was assessed in the initial phone call with the study coordinator. However, it is possible that the speed of the recruitment process and number of lengthy recruitment chains could have benefited from an even more structured process in order to ensure motivation, large number of social ties and further diversity among the seeds. Especially as the results indicate that participants tend to recruit peers that share their characteristics. Therefore, we recommend future studies using webRDS to recruit precariously employed to ensure that the seed recruitment process takes these factors into consideration.

### Limitations

There were several limitations to this study. First, some RDS assumptions were potentially not fulfilled in this study: sampling with replacement and random recruitment. In practice, sampling is commonly done without replacement and earlier samples might therefore have an impact on later samples. This might lead to inaccurate estimates of sampling probabilities which, in turn, could influence the estimates [20]. Further, random recruitment of peers is impossible to enforce and measure among respondents and in many cases selection of peers are based on special reasons [21, 23]. Selective recruitment could result in biased estimates, for instance if network degree or certain variables are associated with recruitment [20, 23].

Second, there are limitations to the study design that could have influenced the successfulness and speed of recruitment. As there is no consensus on the definition of precarious employment and as this population potentially combines several activities and/or employment types, the eligibility criteria used in terms of employment in this study might have been too inflexible. This might have caused eligible participants to be excluded and caused some recruitment chains to die out earlier than necessary. The need to indicate a personal identification number, which is deemed sensitive data, could have limited participation further. In addition, we did not include participants with a so-called co-ordination number, which is given to a foreigner residing in Sweden without a personal identification number, meaning that the study failed to include this group of the precarious worker population. Further, as the study employed an online design it was not possible to completely ensure that participants did not participate repeated times. Incorrect exclusions would result in less power and considerably fewer waves in one recruitment tree than necessary. Finally, it is possible that receiving one reward per recruited peer would have benefited the recruitment as previously done in RDS studies recruiting large samples [27, 35, 36].

### Conclusion

WebRDS satisfactorily recruited a sample of workers with precarious employment that became independent from seeds and from which population estimates could be calculated. The online peer recruitment process produced a diverse sample with a better response rate compared to previous national surveys. Further, previously underrepresented demographic subgroups such as young and males participated to a sufficient extent. Future studies should consider implementing the WebRDS methodology in this population on a national level in order to further our understanding of workers with precarious employment.

## Supporting information

S1 Table Recruitment homophily estimates for eligible recruits and eligible and ineligible recruits. (PDF)

S1 File Data collection instrument in English and Swedish (original language). (PDF)

S2 File Information given to participants and informed consent. (PDF)
