# Supplementary material for: A performance assessment of web-based respondent driven sampling among workers with precarious employment in Sweden
Source: PLoS One. 2019 Jan 10;14(1):e0210183. doi: 10.1371/journal.pone.0210183 (PMC6328181; doi:10.1371/journal.pone.0210183)
Supplement: S1 Table — (PDF) [file pone.0210183.s001.pdf]

S1 Table. Recruitment homophily estimates for eligible recruits and eligible and ineligible recruits

	Eligible recruits (n=358)	Eligible plus ineligible recruits (n=426)
Sex	1.34	1.36
Age	1.65	1.68
Employment type	1.45	1.42
Income	1.27	1.23
Average hours per week	1.35	1.32
