# Supplementary material for: A performance assessment of web-based respondent driven sampling among workers with precarious employment in Sweden
Source: PLoS One. 2019 Jan 10;14(1):e0210183. doi: 10.1371/journal.pone.0210183 (PMC6328181; doi:10.1371/journal.pone.0210183)
Supplement: S1 File — (PDF) [file pone.0210183.s002.pdf]

S1 File. Data collection instrument in English and Swedish (Original language)

Data collection instrument in English

Today's Labour Market and Health

A study from the Unit of Occupational Medicine at Karolinska Institutet

You can get in touch with PREMIS by calling [phone number to study coordinator] (09.00-17.00) or by sending an e-mail to [e-mail to study coordinator] if you have any questions while you are filling in the questionnaire.

You have been invited to participate in this study on today's labor market and health. The purpose of the study is to investigate the associations between various forms of employment and health. The survey includes questions on your health, work, work environment, economy and living conditions.

These are control questions to see if you are eligible for participation.

What is your current form of employment?

If several alternatives fit you, indicate the alternative that fits you the best. If you currently are absent due to sickness, on leave of absence, or absent due to parental leave, indicate the alternative you are absent from.

- Permanently employed
- Involuntarily part-time employed
- Temporarily employed (general temporary employment/project-linked employment/seasonal work/work as substitute)
- Employed by the hour/called when needed
- Self-employed (because it suits me)
- Self-employed (because I can not get an employment)
- Unemployed
- Student
- Taking care of the household
- In early retirement, sickness benefit or activity benefit
- Retirement due to old age
- Trainee

Where do you live and work?

- I live and/or work in Stockholm county
- I neither live nor work in Stockholm county

Information and consent

Today's labour market and health

You have been invited to participate in a survey on today’s labour market and health carried out by the Unit of Occupational Medicine at Karolinska Institutet (KI). Today’s labour market consists of a growing number of precarious employment forms and flexible work arrangements, such as work as substitute, general temporary employment and employment by the hour. There is still too little known about how these forms of employment affect health. The purpose of the study is thus to investigate the associations between precarious employment and common health outcomes in order to increase the knowledge on how these two go together. The survey includes questions on your health, work, work environment, economy and living conditions.

Why you were invited

You have been invited by an acquaintance who also participated in the survey. Since we do not know which residents in Stockholm who work, but do not have a permanent, full-time employment, we rely on participants to invite acquaintances who are in a similar employment situation.

Your answers are important

We still know too little about the impact of precarious employment on health. You belong to a growing social group, and the results from this study may lead to increased knowledge and preventive measures in the future. The survey will be answered by at least 500 participants and the results will be presented at group level - individual responses will not be released. Participation in the survey is voluntary, but your participation is important; it will contribute to the representativeness of the results. You may terminate your participation at any time.

How we use your data

In order to reduce the number of survey questions, and in order to answer our research questions, we will collect complementary data from public registries. You can read more about how we use your data at our webpage.

I understand the information given above and I want to participate

- Yes
- No, I do not want to participate

Please indicate your personal number.

YYYYMMDD-XXXX

Indicate your personal number in the following format: yyyymmdd-xxxx

Your personal identification number and other information

given by you in this study will not be shared with anyone except the researchers. The researchers are bound by the rules of document secrecy and professional secrecy.

Compensation for participation

As a thank you for participating you can choose one out of the following compensations. When you have completed the questionnaire we will send the chosen compensation to you. You will also receive an additional compensation when two of the ones you invited to the study participated. This compensation you can specify in the end of the questionnaire.

- SF gift card (valid for one ticket at SF Bio or Svenska Bio)
- Super gift card, 100SEK (valid for any gift card on presentkorttorget.se)
- I do not want any compensation

Work

How well do you think the following statements describe your current job situation?

Please indicate an answer to all questions.

I have chosen it myself

I think it suits me

I would rather have a full time employment

I would rather have permanent employment

I would rather work with something else

- Not at all
- To a small extent
- To some extent
- To a great extent
- Not applicable

How many hours do you work on average per week?[1]

- Less than 5
- 5-10
- 10-15
- 15-20
- 20-25
- 25-30
- 30-35
- 35-40
- 40-45
- More than 50

IF LESS THAN 35 HOURS ON AVERAGE:

Which is the primary reason that you work part-time?[1]

- Studies
- Sick leave/reduced work capacity
- Looking after children (e.g., parental leave, foster parent)
- I cannot find suitable full-time work/ looking for full-time work
- I have several jobs
- Work is too physically and/or mentally demanding
- I do not want to work full-time
- Other reason

How many jobs (including illicit jobs) do you have?

- None
- 1
- 2
- 3
- 4
- 5
- 6 or more

What is/are your current job/s? [2]

Please describe all your current jobs below. Try to give as detailed a work description as possible. Here are some examples: instead of assistant, write for example purchasing assistant, accounting assistant or advertising assistant. Instead of driver, write for example bus driver, taxi driver or truck driver.

My current job/s

How many jobs have you had the last three years

Count all jobs that lasted for at least two weeks (including illicit jobs). Include current jobs as well.

- None
- One job
- 2-4 jobs
- 5-9 jobs
- 10 or more jobs
- Do not know

What/which job/s have you had the last three years?

Please describe your job/s the last three years below. You do not have to describe your current jobs (indicated in the previous question). Try to give as detailed a work description as possible. Here are some examples: instead of assistant, write for example purchasing assistant,

My job/s the last three years

accounting assistant or advertising assistant. Instead of driver, write for example bus driver, taxi driver or truck driver.

Are you worried about losing your job in the coming year?[2]	- Not at all<br>- To some extent<br>- Quite a lot<br>- Very much
Have you been involuntarily unemployed during the last three years?[4]	- Yes<br>- No
If YES: *roughly, for how long were you involuntarily unemployed the last three years?*[4]<br><br>If you answered yes to the previous question, give an estimate on how many months you were unemployed the last three years.	Number of months
Where do you mainly execute your work?<br><br>If you have several employers, please indicate an answer for the employer you work the most hours for during an average month.	- At my employer<br>- At my employer's client<br>- At my client<br>- At home/home office<br>- Other
We assume that your ability to work, when it is at its best, is valued at 10 points. How many points would you give your current ability to work?[3]<br><br>Please tick the appropriate number. 0 means that you are unable to work, 10 means that your ability to work is at its best right now.	- 1<br>- 2<br>- 3<br>- 4<br>- 5<br>- 6<br>- 7<br>- 8<br>- 9<br>- 10

Work environment

Have you, during the last 12 months, been exposed to/suffered any of the following at work?

Please indicate an answer to all of the questions.

Accident/injury leading to hospital visit?

Accident/injury leading to sick leave?

Violence or threat of violence?

Sexual harassment?

Bullying?

Discrimination due to gender?

Discrimination due to ethnicity?

Discrimination due to age?

- Yes
- No

Compared to permanent employees at your job/s, have you during the last 12 months experienced that you have… Please indicate an answer to all of the questions.

Been given inferior work assignments?

Not been allowed to participate in the same way in activities (staff parties, on-the-job-trainings, planning days etc.)?

Received an inferior work schedule?

Been working more overtime?

Received lower salary for the same work assignments?

- Never
- Rarely
- Sometimes
- Often
- Always
- Not applicable

Requirements and influence[3]

If you have more than one employer, please indicate the employer you work the most hours for during an average month.

Do you have the opportunity to determine your work pace?

Is your work so stressful that you do not have time to talk or even think about something other than work?

Can you take short breaks at virtually any time?

- Nearly all the time
- About 3/4 of the time
- Half the time
- About 1/4 of the time
- About 1/10 of the time
- No, not at all

Does work require your full attention and concentration?

Are you required to lift at least 15 kg at a time several times per day?[3]

- Every day
- A couple of days per week
- One day per week
- A couple of days per month
- Not at all/rarely in the last 3 months

Terms of employment[5]

The questions in this section concern the job where you work the most hours during an average month.

How long is your current employment contract valid?

If you have more than one employer, please indicate the employer you work the most hours for during an average month.

- Indefinitely
- 2 years or more
- 1 year or more
- 6 months or more
- 3 months or more
- 1 month or more
- Less than 1 month
- Do not have a contract
- Do not know

How long have you been working for the same employer?

If you have more than one employer, please indicate the employer you work the most hours for during an average month.

- Less than 1 month
- 1 month to less than 3 months
- 3 months to less than 6 months
- 6 months to less than 1 year
- 1 year to less than 2 years
- 2 years to less than 5 years
- 5 years or more
-

Approximately how much do you earn per month after taxes?

Add the amount for your salary after tax deductions + salary for illicit work + any tips, during an average month.

- 3000 SEK or less
- Between 3001 and 6000 SEK
- Between 6001 and 9000 SEK
- Between 9001 and 12 000 SEK
- Between 12 001 and 15 000 SEK
- Between 15 001 and 18 000 SEK
- Between 18 001 and 21 000 SEK
- Between 21 001 and 24 000 SEK
- Between 24 001 and 27 000 SEK
- Between 27 001 and 30 000 SEK
- More than 30 000 SEK

- No answer

How often does your current salary allow you to...

…Cover you daily basic needs?

…Cover unforeseen expenses of significance?

- Always
- Often
- Sometimes
- Rarely
- Never

How were your working hours settled for your current job?

Indicate the alternative that fits you best. If you have more than one employer, please indicate the employer you work the most hours for during an average month.

- My working hours are in accordance with Swedish law and the collective agreement
- My employer decided my working hours
- My working hours are the result of an agreement between me and my manager
- My working hours are the result of an agreement within my work team
- Do not know
- Not applicable. I work project-based

How was the salary settled for your current job?

Indicate the alternative that fits you best.

If you have more than one employer, please indicate the employer you work the most hours for during an average month.

- My salary is accordance with the collective agreement
- My salary was set by my employer
- My salary is the result of an agreement between me and my manager
- My salary is the result of an agreement in my work team
- Do not know

Indicate how often, at your employer...

If you have more than one employer, please indicate the employer you work the most hours for during an average month. Please indicate an answer to all of the questions.

You feel afraid to demand better working conditions

You are defenceless towards unfair treatment by your superiors

- Always
- Often
- Sometimes

- Rarely
- Never

You feel afraid of being fired if you do not comply with everything your employer asks of you

You are treated in an authoritarian manner

You are made to feel easily replaceable

Do you have the right to any of the following?
Please indicate an answer to all of the questions.

Parental leave

Retirement due to old age

Unemployment insurance fund (A-kassa)

Severance pay in the event of termination

Sickness benefit

- Yes
- No
- Do not know

How often, in the organisation where you work, are you able to exercise the following rights?

If you have more than one employer, please indicate the employer you work the most hours for during an average month. Please indicate an answer to all of the questions.

Take the weekend off/ weekly rest without problem

Take vacation days without problem

Take a day off for family reasons without problem (care of a sick child, care of a sick relative etc.)

Take a day off for personal reasons without problem

Go on sick leave without problem

Go to the doctor when needed

- Always
- Often
- Sometimes
- Rarely
- Never

Life situation

During the last 12 months, have you had difficulty in managing the regular expenses for food, rent, bills etc.?[2]

- No
- Yes

- Yes, more than once

If you should suddenly find yourself in an unforeseen situation where you had to acquire 14 000 SEK in one week, would you manage it[4]

- Yes
- No

Considering your current life situation, how often do you experience that...

Please indicate an answer to all questions.

You decline social activities because you have to say yes to work?

You decline social activities because you do not know if you can afford it?

You say yes to work because you are afraid you will not be contacted next time?

You find it difficult to piece several jobs or projects together?

You avoid talking about your work situation in social contexts?

- Always
- Often
- Sometimes
- Never

Health

The questions in this section regards your health.
You have now completed more than 80% of the questionnaire.

How would you describe your health in general? Is it...[2]

- Very good
- Good
- Fair
- Poor
- Very poor

During the past three months after work had pain in... [3]

… upper back or neck?

… lower back?

- Every day
- A couple of days per week
- One day per week
- A couple of days per month

… shoulders or arms?

… wrists or hands?

… hips, legs, knees or feet?

- Not at all/rarely in the last three months

How many times during the past 12 months have you worked, even though you really should have not worked given your medical condition?[3]

- Never
- Once
- Two to three times
- Four times or more

GHQ-12[2]. Not spelled out due to copyright reasons.

Background questions

What is your age? Indicate your age in whole years.

Age in years

Indicate the postal code of the address where you sleep most weekdays

Postal code (xxxxx)

Indicate the postal code of the address where you are registered

Postal code (xxxxx)

Invitations and recruitment

After this section we will ask you to invite a couple of your friends and acquaintances that also work but doesn't have permanent employment to take part in the study. First, we ask you to answer the questions below concerning your social network in order to be able to evaluate if this type of study is a good way to reach the group of employed with insecure employment.

How many people whom are also precarious workers (working but without a permanent, full time employment), older than 18 years, could you invite to this study via internet if the invitations were not limited to four?

(You will not be asked to invite all of those you indicate here)

Number

Of these, how many are in the same age group as yourself?<br><br>With same age group we mean 5 years younger to 5 years older (+/- 5 years) than yourself.	- All or close to all (80-100%)<br>- More than half (60-80%)<br>- Half (40-60%)<br>- Less than half (20-40%)<br>- A few (0-20%)<br>- None<br>- Don't know
Of these, how many live in the same municipality as you?	- All or close to all (80-100%)<br>- More than half (60-80%)<br>- Half (40-60%)<br>- Less than half (20-40%)<br>- A few (0-20%)<br>- None<br>- Don't know
What is your relationship with the person who invited you to this study?	- He/she is a relative/member of the family<br>- He/she is my current partner<br>- He/she is an ex-partner<br>- He/she is a close friend<br>- He/she is a friend<br>- He/she is an acquaintance<br>- He/she is a co-worker<br>- I was invited by the research group<br>- He/she is stranger to me (I have not communicated with him/her before I got the invitation to this study)<br>- Don't want to answer

Recruitment

Your compensation

Thank you for your participation! You have now completed the survey, and what is left for you to do is to specify the compensations you would like to receive and which of your friends and acquaintances you would like to invite. Specify the compensation you chose in the beginning of the survey below. An additional compensation will be sent to you when two of your invited acquaintances responded to the questionnaire. Compensations will be sent via e-mail within two working days.

After you have chosen your compensations, click 'confirm' in order to move on the invitation-section.

For participating in the survey I wish to receive...<br><br>*When two of my friends complete the survey I wish to receive...*	- SF gift card (valid for one ticket at SF Bio or Svenska Bio)<br>- Super gift card, 100SEK (valid for any gift card on presentkorttorget.se)<br>- I do not want any compensation

Invite participants

You can invite a maximum of 4 participants. You can choose how you want to invite them in accordance with the options below.

The ones you invite must work but not have a permanent, full time, employment (for example general temporary employment/project-linked employment/employed by the hour, be involuntarily part-time employed or involuntarily self-employed) work and/or live in Stockholm county and be 18-65 years in order to be able to participate.

You can see your invitations at the bottom of this page. When you have completed the questionnaire you can use the participation link that was sent to your e-mail in order enter the survey again and see if the ones you invited participated. If they did not participate yet, please remind them to do so. When at least two of the ones you invited participated in the survey, we will send you the second compensation you chose.

Thank you for participating in the study!

Data collection instrument in Swedish (original language)

Dagens arbetsmarknad och hälsa

En studie från Enheten för Arbetsmedicin på Karolinska Institutet.

Du kan kontakta PREMIS på telefonnummer [telefonnummer till studiekoordinator] (09.00-17.00) eller på [e-post till studiekoordinator] om du har några frågor under tiden du fyller i enkäten.

Du har blivit inbjuden att delta i denna enkät om dagens arbetsmarknad och hälsa. Syftet med studien är att undersöka sambandet mellan olika anställningsformer och hälsa. I enkäten ställs frågor om din hälsa, arbete, arbetsmiljö, ekonomi och livssituation.

Detta är kontrollfrågor för att se om undersökningen är för dig.

Vilket av följande alternativ passar bäst in på dig?

Om flera olika alternativ passar in anger du det som du tycker passar bäst. Om du är arbetslös, anger du arbetssökande som alternativ. Om du är sjukskriven, tjänstledig eller föräldraledig anger du det alternativ som du är ledig/borta från.

- Tillsvidareanställning (fast anställning)
- Ofrivilligt deltidsanställd
- Visstidsanställning/ projektanställning/ säsongsanställning/vikariat
- Behovs-/timanställning
- Egen företagare (för att det passar mig)
- Egen företagare (för att jag inte får en anställning)
- Arbetssökande
- Studerande
- Hemarbetande/sköter hushållet
- Förtidspensionär/innehar sjukersättning eller aktivitetsersättning
- Ålders- eller avtalspensionär
- Praktikant

Var bor och arbetar du?

- Jag bor och/eller arbetar i Stockholms län
- Jag varken bor eller arbetar i Stockholms län
-

Information och samtycke

Dagens arbetsmarknad och hälsa

Du har blivit inbjuden att delta i en denna enkät om dagens arbetsmarknad och hälsa. Undersökningen genomförs av Enheten för arbetsmedicin på Karolinska Institutet. Arbetsmarknaden består idag av en växande del osäkra anställningsvillkor och flexibla anställningar, såsom vikariat, visstids- och behovsanställningar. Vi vet ännu för lite om hur dessa anställningsformer påverkar hälsan. Enkätens syfte är att undersöka sambandet mellan osäkra anställningar och vanliga hälsoutfall för att öka kunskapen om hur dessa hänger ihop. I enkäten ställs frågor om din hälsa, arbete, arbetsmiljö, ekonomi och livssituation.

Därför har du blivit inbjuden

Du har blivit inbjuden av en bekant som också svarat på enkäten. Eftersom vi inte vet vilka i Stockholm som har arbete men som inte har en fast heltidsanställning, förlitar vi oss på att deltagare bjuder in bekanta som är i en liknande anställningssituation.

Dina svar är viktiga

Vi vet fortfarande för lite om vilken påverkan osäkra anställningsvillkor har på hälsan. Du tillhör en växande samhällsgrupp och resultaten från studien kan leda till ökad kunskap och förebyggande insatser i framtiden. Enkäten kommer att besvaras av minst 500 deltagare och resultaten kommer att presenteras på gruppnivå – enskilda svar lämnas inte ut. Det är frivilligt att delta i undersökningen, men din medverkan är viktig. Den bidrar till att resultatet blir representativt. Du kan när som helst avbryta din medverkan.

Hur lämnade uppgifter används

För att minska antalet enkätfrågor och för att få svar på våra forskningsfrågor kommer kompletterande uppgifter även att tas från register. Du kan läsa mer om hur dina uppgifter används på vår hemsida.

Jag är införstådd med informationen ovan och vill delta i studien.

- Ja
- Nej, jag vill inte delta

Var god ange ditt personnummer.

ÅÅÅÅMMDD-XXXX

Ange ditt personnummer med 12 siffror i formatet ååååmmdd-xxxx

Personnumret och annan information angiven i den här studien kommer inte att delges någon annan än forskarna. Forskarna omfattas av reglerna om handlingssekretess och tystnadsplikt.

Ersättning för deltagande

Som tack för din medverkan kan du välja en av följande ersättningar. När du fyllt i enkäten kommer vi att skicka vald ersättning till dig. Du kommer även att motta en ersättning när två av de du bjudit in till studien deltagit. Denna ersättning kan du specificera i slutet på enkäten.

- SF-presentkort
- Superpresenkort 100 kr (gäller på valfritt presentkort på presentkorttorget.se)
- Jag önskar ingen ersättning

Arbete

Hur väl anser du att följande påståenden beskriver din nuvarande arbetssituation?

Var god besvara alla frågor.

Jag har valt den själv

Jag tycker den passar mig

Jag skulle hellre ha en heltidstjänst

Jag skulle hellre ha fast anställning

Jag skulle hellre jobba med något annat

- Inte alls
- I liten utsträckning
- I viss utsträckning
- I hög utsträckning
- Ej relevant

Hur många timmar arbetar du i genomsnitt en vanlig vecka?

- Färre än 5
- 5-10
- 10-15
- 15-20
- 20-25
- 25-30
- 30-35
- 35-40
- 40-45
- Fler än 50

OM FÄRRE ÄN 35 TIMMAR I VECKAN I GENOMSNITT:

Vad är främst anledningen till att du arbetar deltid?[1]

- Studier
- Sjukdom/nedsatt arbetsförmåga
- Vård av barn (Föräldraledighet/fosterförälder)

- Jag kan inte hitta heltidsarbete/ söker efter heltidsarbete
- Jag har flera jobb
- Orkar inte – arbetet är för fysiskt och/eller psykiskt krävande
- Jag vill inte arbeta heltid
- Annat skäl

Hur många jobb (inklusive svartjobb) har du för tillfället?

- Inget
- 1
- 2
- 3
- 4
- 5
- 6 eller fler

Vilket/Vilka är ditt/dina nuvarande arbete/n?[2]

Var god beskriv alla dina nuvarande arbeten nedan. Försök att lämna en så detaljerad yrkesbeskrivning som möjligt. Här följer några exempel: istället för att skriva assistent, skriv till exempel inköpsassistent, redovisningsassistent eller reklamassistent. Istället för att skriva chaufför, skriv till exempel busschaufför, taxichaufför eller lastbilschaufför.

Mitt/mina nuvarande arbete/n

Hur många arbeten har du haft de senaste tre åren?

Räkna alla arbeten som varat minst två veckor (även svartjobb). Räkna även in dina nuvarande arbeten.

- Inget
- Ett arbete
- 2-4 arbeten
- 5-9 arbeten
- 10 eller fler arbeten
- Vet ej

Vilket/vilka har varit ditt/dina arbete/n de senaste tre åren?

Beskriv ditt/dina arbete/n de senaste tre åren nedan. Du behöver inte beskriva dina nuvarande arbeten (indikerade i föregående fråga). Försök att lämna en så detaljerad yrkesbeskrivning som möjligt. Här följer några exempel: istället för att skriva assistent, skriv till exempel inköpsassistent, redovisningsassistent eller reklamassistent. Istället för att skriva chaufför, skriv till exempel busschaufför, taxichaufför eller lastbilschaufför.

Mitt/mina arbete/n de senaste tre åren

Är du orolig för att bli arbetslös inom det närmaste året?[2]	- Inte alls orolig<br>- Inte särskilt orolig<br>- Ganska orolig<br>- Mycket orolig
Har du varit ofrivilligt arbetslös under de senaste tre åren?[4]	- Ja<br>- Nej
OM JA: *Ungefär hur lång tid har du sammanlagt varit arbetslös under de senaste tre åren?*[4]<br><br>Om du svarat ja på föregående fråga, uppskatta ungefär hur många månader totalt du varit arbetslös de senaste tre åren.	Antal månader
Var utför du i huvudsak ditt arbete?<br><br>Om du har fler arbetsgivare anger du svaret för den arbetsgivare du arbetar för flest timmar en genomsnittlig månad.	- Hos min arbetsgivare/uppdragsgivare<br>- Hos min arbetsgivares/uppdragsgivares kund<br>- Hos min kund<br>- Hemma/eget kontor<br>- Annat
Vi antar att din arbetsförmåga, då den är som bäst, värderas med 10 poäng. Vilket poängtal skulle du ge din nuvarande arbetsförmåga?[3]<br><br>Välj lämplig siffra. 0 betyder att du inte alls kan arbeta, 10 betyder att du arbetar som allra bäst just nu.	- 1<br>- 2<br>- 3<br>- 4<br>- 5<br>- 6<br>- 7<br>- 8<br>- 9<br>- 10

Arbetsmiljö

Har du under de senaste 12 månaderna blivit utsatt/råkat ut för något av följande på jobbet?<br><br>Var god besvara alla frågor.<br><br>Olycka/skada som lett till sjukvårdsbesök?<br><br>Olycka/skada som lett till sjukskrivning?	- Ja<br>- Nej

Våld eller hot om våld?

Sexuella trakasserier?

Mobbning?

Diskriminering på grund av kön?

Diskriminering på grund av etnisk tillhörighet?

Diskriminering på grund av ålder?

Jämfört med fast anställda på ditt/dina arbeten, har du under de senaste 12 månaderna upplevt att du...

Var god besvara alla frågor.

Getts sämre arbetsuppgifter?

Inte fått delta på samma sätt i aktiviteter (personalfester, utbildningar, planeringsdagar etc.)?

Fått sämre schema?

Arbetat mer övertid?

Fått sämre lön för samma arbetsuppgifter?

- Aldrig
- Sällan
- Ibland
- Ofta
- Alltid
- Ej relevant

Krav och kontroll[3]

Om du har flera arbetsgivare anger du den arbetsgivare du arbetar för flest timmar en genomsnittlig månad.

Har du möjlighet att själv bestämma din arbetstakt?

Har du så stressigt att du inte hinner prata om eller ens tänka på något annat än arbetet?

Kan du ta korta pauser i stort sett när som helst?

Kräver arbetet hela din uppmärksamhet och koncentration?

- Nästan hela tiden
- Ungefär 3/4 av tiden
- Halva tiden
- Ungefär 1/4 av tiden
- Ungefär 1/10 av tiden
- Nej, inte alls

Måste du flera gånger om dagen lyfta minst 15 kg åt

- Varje dag
- Ett par dagar per vecka

gången?[3]

- En dag per vecka
- Ett par dagar per månad
- Inte alls/sällan de sista 3 månaderna

Anställningsvillkor[5]

Frågorna i detta avsnitt gäller det arbete där du jobbar flest timmar en genomsnittlig månad.

Hur länge gäller ditt nuvarande kontrakt?

Om du har flera arbetsgivare anger du den arbetsgivare du arbetar för flest timmar en genomsnittlig månad.

- Tillsvidare
- 2 år eller mer
- 1 år eller mer
- 6 månader eller mer
- 3 månader eller mer
- 1 månad eller mer
- Kortare än 1 månad
- Har inget kontrakt
- Vet ej

Hur länge har du jobbat för samma arbetsgivare/uppdragsgivare?

Om du har flera arbetsgivare anger du den arbetsgivare du arbetar för flest timmar en genomsnittlig månad.

- Mindre än 1 månad
- 1 månad till mindre än 3 månader
- 3 månader till mindre än 6 månader
- 6 månader till mindre än 1 år
- 1 år till mindre än 2 år
- 2 år till mindre än 5 år
- 5 år eller mer

Ungefär hur mycket tjänar du per månad netto (efter skatt)?

Lägg ihop summan för din vita lön efter skatt + svart lön + eventuell dricks, en genomsnittlig månad.

- 3 000 kr eller mindre
- Mellan 3 001 och 6 000 kr
- Mellan 6 001 och 9 000 kr
- Mellan 9 001 och 12 000 kr
- Mellan 12 001 och 15 000 kr
- Mellan 15 001 och 18 000 kr
- Mellan 18 001 och 21 000 kr
- Mellan 21 001 och 24 000 kr
- Mellan 24 001 och 27 000 kr
- Mellan 27 001 och 30 000 kr
- Mer än 30 000 kr
- Inget svar

Hur ofta tillåter din nuvarande lön dig att...

- Alltid

Täcka dina dagliga grundläggande behov?

Täcka oförutsedda utgifter av betydelse?

- Ofta
- Ibland
- Sällan
- Aldrig

Hur bestämdes arbetstiderna för ditt nuvarande arbete?

Välj det alternativ som stämmer bäst in på dig. Om du har flera arbetsgivare anger du den arbetsgivare du arbetar för flest timmar en genomsnittlig månad.

- De följer lag och kollektivavtalet
- De bestämdes av arbetsgivaren
- De var en överenskommelse mellan mig och min chef
- De var en överenskommelse i mitt arbetslag
- Vet ej
- Ej relevant, arbetar uppdragsbaserat

Hur bestämdes lönen för ditt nuvarande arbete?

Välj det alternativ som stämmer bäst in på dig. Om du har flera arbetsgivare anger du den arbetsgivare du arbetar för flest timmar en genomsnittlig månad.

- Den följer kollektivavtalet
- Den bestämdes av arbetsgivaren
- Den var en överenskommelse mellan mig och min chef
- Den var en överenskommelse i mitt arbetslag
- Vet ej

Ange hur ofta hos din arbetsgivare som...

Om du har flera arbetsgivare anger du den arbetsgivare du arbetar för flest timmar en genomsnittlig månad. Var god besvara alla frågor.

Du är rädd för att kräva bättre arbetsvillkor

Du är försvarslös mot orättvis behandling från överordnande

Du är rädd för att få sparken om du inte gör allt arbetsgivaren ber om

Du blir behandlad auktoritärt

De får dig att känna dig lätt utbytbar

- Alltid
- Ofta
- Ibland
- Sällan
- Aldrig

Har du rätt till något av följande?

Var god besvara alla frågor.

Föräldraledighet<br>Ålderspension<br>A-kassa<br>Avgångsvederlag vid uppsägning<br>Sjukersättning/sjukpenning	- Ja<br>- Nej<br>- Vet ej
Hur ofta i den organisation där du arbetar kan du utöva följande rättigheter?<br>Om du har flera arbetsgivare anger du den arbetsgivare du arbetar för flest timmar en genomsnittlig månad.<br>Var god besvara alla frågor.<br>Ta helg/veckovila utan problem<br>Ta semesterdagar utan problem<br>Ta en ledig dag av familjeskäl utan problem (vård av sjukt barn, vård av sjuk anhörig etc.)<br>Ta en ledig dag av personliga skäl utan problem<br>Sjukskriva dig utan problem<br>Gå till läkare när du behöver	- Alltid<br>- Ofta<br>- Ibland<br>- Sällan<br>- Aldrig

Livssituation

Har du under de senaste 12 månaderna haft svårigheter att klara de löpande utgifterna för mat, hyra, räkningar, m.m.?[2]	- Nej<br>- Ja, vid ett tillfälle<br>- Ja, vid flera tillfällen
Om du plötsligt skulle hamna i en oförutsedd situation där du på en vecka måste skaffa fram 14 000 kr, skulle du klara det?[4]	- Ja<br>- Nej
Om du tänker på din nuvarande livssituation, hur ofta upplever du att...	

Var god besvara alla frågor.

Du tackar nej till sociala aktiviteter på grund av att du måste tacka ja till jobb?	- Alltid<br>- Ofta<br>- Sällan<br>- Aldrig
Du tackar nej till sociala aktiviteter på grund av att du inte vet om du kommer ha råd?	
Du tackar ja till jobb för att du är rädd att inte bli kontaktad nästa gång?	
Du har svårt att pussla ihop flera jobb eller uppdrag med varandra?	
Du undviker att berätta om din arbetssituation i sociala sammanhang?	

Hälsa

Frågorna i denna sektion handlar om din hälsa.
Du har nu genomfört mer än 80% av enkäten

Hur bedömer du ditt allmänna hälsotillstånd? Är det...[2]

- Mycket gott
- Gott
- Någorlunda
- Dåligt
- Mycket dåligt

Har du under de senaste tre månaderna efter arbetet haft ont i... 3

Var god besvara alla frågor.

... övre delen av ryggen eller nacken?	- Varje dag<br>- Ett par dar per vecka<br>- En dag per vecka<br>- Ett par dar per månad<br>- Inte alls/sällan de sista tre månaderna
... nedre delen av ryggen?	
... axlar eller armar?	
... handleder eller händer?	
... höfter, ben, knän eller fötter?	

Hur många gånger under de senaste 12 månaderna har det hänt att du arbetat trots att du med tanke på ditt

- Aldrig
- En gång

hälsotillstånd egentligen borde ha låtit bli?[3]

- Två till tre gånger
- Fyra gånger eller mer

GHQ-12[2]. Ej inkluderad på grund av upphovsrättsliga skäl.

Bakgrundsfrågor

Vad är din ålder? Ange hela år.

Ålder i år

Ange postnummer till den adress där du sover de flesta vardagsnätterna

Postnummer (xxxxx)

Ange postnummer till den adress där du är skriven/folkbokförd

Postnummer (xxxxx)

Inbjudningar och vidarerekrytering

Efter denna sektion kommer vi att be dig bjuda in upp till fyra vänner och bekanta som också arbetar som huvudsaklig sysselsättning, men som inte har en fast heltidsanställning, till att delta i studien. Innan dess ber vi dig besvara nedanstående om ditt sociala nätverk för att kunna utvärdera om den här typen av studier på internet är ett bra sätt att nå ut till gruppen anställda med en osäker anställning.

Hur många med osäkra anställningar (som huvudsakligen arbetar, men som inte har fast heltidsanställning), äldre än 18 år, skulle du kunna bjuda in via internet till den här studien om inte inbjudningarna var begränsade till fyra?

(Du kommer inte behöva bjuda in alla du uppger här)

Antal

Av dessa, hur många är i samma åldersgrupp som du själv?

Med samma åldersgrupp menar vi 5 år yngre till 5 år äldre (+/- 5 år) än du själv.

- Alla eller nästan alla (80-100%)
- Mer än hälften (60-80%)
- Hälften (40-60%)
- Mindre än hälften (20-40%)
- Ett fåtal (0-20%)
- Inga
- Vet ej

Av dessa, hur många bor i samma kommun som du själv?

- Alla eller nästan alla (80-100%)
- Mer än hälften (60-80%)
- Hälften (40-60%)

- Mindre än hälften (20-40%)
- Ett fåtal (0-20%)
- Inga
- Vet ej

Vilken är din relation till personen som bjöd in dig till den här studien?

Indikera ett eller två alternativ nedan.

- Hon/hon är en familjemedlem/släkting
- Han/hon är min nuvarande partner
- Han/hon är en före detta partner
- Han/hon är en vän som jag kan prata om personliga ämnen med
- Han/hon är en vän, men ingen jag kan prata om personliga ämnen med
- Han/hon är en bekant
- Han/hon är en arbetskamrat
- Jag blev inbjuden av forskargruppen
- Han/hon är okänd för mig (jag har inte kommunicerat med honom/henne innan jag blev inbjuden till den här studien)
- Vill inte svara

Rekrytering

Din ersättning

Tack för ditt deltagande! Du har nu fyllt i hela enkäten och det som återstår för dig är att specificera vilka ersättningar du önskar och vilka av dina vänner och bekanta du vill bjuda in till studien. Indikera ersättningen du valde i början av studien nedan. Ytterligare en ersättning skickas till dig efter att två av de du bjudit in till studien besvarat enkäten, denna kan du också specificera nedan. Ersättningar skickas via e-post inom två arbetsdagar.

Efter att du valt ersättning, klicka på 'gå vidare' för att gå vidare till inbjudningarna.

Som ersättning för mitt deltagande i studien vill jag gärna få...

När minst två av mina inbjudna vänner deltagit vill jag få...

- SF-presentkort
- Superpresenkort 100 kr (gäller på valfritt presentkort på presentkorttorget.se)
- Jag önskar ingen ersättning

Bjud in deltagare

Då vi inte vet vilken typ av anställning Stockholmare har idag bygger studien på att deltagare bjuder in vänner och bekanta som arbetar men som inte har en fast heltidstjänst till att delta. Varenda deltagare är viktig för oss. Därför ber vi dig bjuda in upp till 4 personer.

De du bjuder in måste huvudsakligen arbeta men inte ha en fast heltidsanställning (t ex. ha en visstidsanställning, vikariat, ofrivillig deltidsanställning eller ofrivillig egen företagare), bo och/eller arbeta i Stockholms län och vara 18-65 år, för att kunna delta. Heltidsstudenter kan inte delta i studien.

Du kan se dina inbjudningar längst ner på sidan. När du slutfört enkäten kan du använda din deltagarlänk som du fått skickad till dig för att gå in och se om de du bjudit in besvarat enkäten. När två av de du bjudit in genomfört enkäten skickar vi den andra ersättningen du valt.
Tack för att du deltagit i studien!

Footnotes

1. Item taken from the Swedish work force survey of 2015 [In Swedish]. Link: https://www.scb.se/contentassets/c12fd0d28d604529b2b4ffc2eb742fbe/am0401_do_2015_150421.pdf

2. Item taken from the Swedish public health survey of 2015 [In Swedish]. Link: https://www.folkhalsomyndigheten.se/contentassets/840c39c076eb48bc8a1cbfdfdfd01a22/formular-nationella-folkhalsoenkaten-2015.pdf

3. Item taken from the Swedish work environment survey of 2015 [In Swedish]. Link: https://www.scb.se/contentassets/a2ad4e9c34774bb096179640bd44a6f1/am0501_do_2015.pdf

4. Item taken from the public health survey of Scania 2010 [In Swedish]. Link not available.

5. Item taken from the Employment Precariousness Scale 2010.
Vives A, Gonzalez F, Moncada S, Llorens C, Benach J. Measuring precarious employment in times of crisis: the revised Employment Precariousness Scale (EPRES) in Spain. Gaceta sanitaria / SESPAS. 2015;29(5):379-82.
