# Supplementary material for: A performance assessment of web-based respondent driven sampling among workers with precarious employment in Sweden
Source: PLoS One. 2019 Jan 10;14(1):e0210183. doi: 10.1371/journal.pone.0210183 (PMC6328181; doi:10.1371/journal.pone.0210183)
Supplement: S2 File — (PDF) [file pone.0210183.s003.pdf]

S2 File. Information given to participants and informed consent

Today's Labour Market and Health

You have been invited to participate in a survey on today's labour market and health carried out by the Unit of Occupational Medicine at Karolinska Institute (KI). Today's labour market consists of a growing number of precarious employment forms and flexible work arrangements, such as work as substitute, general temporary employment and employment by the hour. There is still too little known about how these forms of employment affect health. The purpose of the study is thus to investigate the associations between the state of the labour market and common health outcomes in order to increase the knowledge on how these two go together. The survey includes questions on your health and potential illnesses, work and work environment, economy, housing, and living conditions.

Why you were invited

You have been invited by an acquaintance who also participated in the survey. Since we do not know which residents in Stockholm that are employed in general temporary employment or by the hour, or who are self-employed, we rely on participants to invite acquaintances who are in a similar employment situation.

Your answers are important

We still know too little about the impact of precarious employment on health. You belong to a growing social group, and the results from this study may lead to increased knowledge and preventive measures in the future. The survey will be answered by at least 500 participants and the results will be presented at group level - individual responses will not be released. Participation in the survey is voluntary, but your participation is important; it will contribute to the representativeness of the results. You may terminate your participation at any time.

How we use your data

In order to reduce the number of survey questions, and in order to answer our research questions, we will collect complementary data from public registries. Since an individual's health is affected by heredity and family relationships, we will also collect information on parents, siblings, and children. Information on gender, age, social background, family relationships, marital status, and country of birth, citizenship, housing area, education, occupation, income, social benefits and benefits during sick leave will be collected from Statistics Sweden (Statistiska Centralbyrån; SCB). Information on hospital care, pregnancy and birth, and use of pharmaceutical products will be collected from The National Board of Health and Welfare and the Swedish Association of Local Authorities (Socialstyrelsen) and Regions (Sveriges Kommuner och Landsting). Stockholm County Council (Stockholms läns landsting) will provide information on care and treatment results, and the Swewdish Social Insurance Agency (Försäkringskassan) will provide diagnoses in connection with any sick leave or disablement pension ("sjuk- och aktivitetsersättning"). Information from any military enrolment will be collected from the Swedish Defence Recruitment Agency (Rekryteringsmyndigheten) or Military Archives (Krigsarkivet). The City of Stockholm will provide data on the levels of noise and air pollution in the housing area. When responding to the survey questions, you also agree to the use of this registry information. After completed processing at Statistics Sweden, all personal data will be removed, and the material will be handed over to Karolinska Institutet for further processing and analysis. Information from the survey might be used by several researchers, but release of any data will always be carried out restrictively, and after careful scrutiny. This research and these statistics are also covered by secrecy of statistics (see below). Since the health of the study participants will be followed over time, we hope that the study will provide important answers to the causes to various illnesses and how these can be prevented. Thus, a new survey may be sent out in a few years. Participation is always voluntary and you will decide at each occasion if you wish to participate. To be able to send out new surveys, and in order to make it possible to supplement new surveys with current registry information, we will keep a record of participants. This information will be saved separately from survey responses and other registry

information.

Protection of Survey Responses

Your survey responses will not be identifiable when the results from the study are published. Any information provided to us is protected by secrecy in accordance with “24 kap. 8 §” in the Information and Secrecy Act (“offentlighets- och sekretesslagen”, 2009:400). Employees at KI and SCB working with the study are bound by the rules of document secrecy and professional secrecy. Rules for the processing of personal data are also given in the Personal Data Act (“personuppgiftslagen”, 1998:204), as well as in the Official Statistics Act (“lagen om den offentliga statistiken”, 2001:99) and the Regulation on Official Statistics (“förordningen om den offentliga statistiken”, 2001:100).

Personal data

KI is the controller of personal data and responsible for the processing of information carried out by KI. SCB is the controller of personal data and responsible for the processing of personal data carried out by SCB. Any individual with a personal record has the right to once per calendar year receive information free of charge by a so called “registerutdrag” (record of the registry) on the personal data handled by the SCB and by KI. A request for a “registerutdrag” must be made in written form and must be signed by the person whom the record concerns. For more information, go to www.scb.se/pul. Should you find that KI or the SCB has treated your personal data in a way that violates the Personal Data Act, you may request to have your personal data corrected, blocked or erased.
